# Creation of a new frailty scale in primary care: the Zulfiqar Frailty Scale

**DOI:** 10.22088/cjim.13.2.425

**Published:** 2022

**Authors:** Abrar-Ahmad Zulfiqar

**Affiliations:** 1 Internal Medicine Unit, Clinique Médicale B, University Hospital of Strasbourg, 67000 Strasbourg, France

**Keywords:** Frailty, Elderly, primary care, Zulfiqar Frailty Scale (ZFS)

## Abstract

**Background:**

Preventing dependency is a public health objective. We want to evaluate the ability of the “Zulfiqar Frailty Scale” (ZFS) tool to detect frailty as defined by Fried's criteria among a group of patients aged 75 and older.

**Methods:**

Prospective study conducted in Poitou-Charentes (France) for 12 months on patients aged 75 and over and considered autonomous in terms of the ADL scale. To be eligible, the patients could not reside in a nursing home and needed an ADL score of 4 or higher.

**Results:**

Among the group of 200 patients (with a mean age of 81.4 years, +/- 4.82), the prevalence of frailty according to Fried's criteria was 32.5%. The prevalence of frailty according to the “Zulfiqar Frailty Scale” tool was 35.0% and all items except home confinement were significantly associated with frailty. With this tool, the threshold for identifying frailty was 3 out of 6 criteria. It was quick (average completion time of 2 minutes and 2 seconds) with a sensitivity score of 88.0% and a negative predictive value of 91.0%.

**Conclusion:**

The “Zulfiqar Frailty Scale” tool measures frailty just as effectively as Fried’s criteria, with sensitivity and negative predictive values no lower than the latter.

Preventing dependency is a public health objective. Frailty can be used to predict the risk of dependency, falling, hospitalization, and death. General practitioners would be the best choice for identifying frailty, but it is hard to do this in current practice with validated tools (1-2). Ideally, as with any screening tool, these frailty detection scores should be sensitive to identify as many frail individuals as possible, but also specific to avoid performing a standardized geriatric test that is not very useful or efficient for those who are not frail. These scores must be validated by comparing them with benchmark tools. Finally, to be useful, a frailty detection score must be relevant for all health practitioners (whether or not they are doctors), which means we need a simple, reproducible set of criteria so we can avoid a lengthy period of instruction beforehand. A tool with these characteristics should be quick to use. There is no consensus regarding frailty diagnostic criteria. The prevalence of frailty depends on the tool used. In the European SHARE study, the prevalence of frailty varied from 6% to 43% depending on the eight tools used (1). These tools were validated by international cohort studies for diagnosing frailty, but appear difficult to use in general medical practice. Therefore, we have developed a tool for identifying frailty in general medicine for independent subjects over 65 years old that is intended to be quick and easy to use. It takes into account various factors related to frailty risk (social, cognitive, nutritional, falls, and iatrogenic). The objective of this preliminary task is to determine the performance of the “Zulfiqar Frailty Scale (ZFS)” tool to detect frailty (defined by the Fried criteria) in this ambulatory population. Its secondary objectives are to evaluate the feasibility and acceptability of our “ZFS” tool in the setting of a general medicine consultation and to evaluate the prevalence of each item of the “ZFS” composite tool in a population of older subjects who see general practitioners.

## Methods

*Study type* *: Prospective study conducted in 4 general practices in the Charente region of France. Patients were selected to participate in the study over a period of 12 months between June 1, 2019 and May 30, 2020.* *Frailty screening with the “Zulfiqar Frailty Scale” (ZFS) tool* *: The score was calculated by way of six indicators that measured the main functions of an elderly person (3-4-5-6) in terms of their geriatric relevance as defined by the scientific literature. A point was assigned for each positive indicator (maximum score = 6). See* table 1 *for details.*

**Table 1.** Zulfiqar Frailty Scale (ZFS)

Is there a weight loss greater than or equal to 5% in 6 months?	Yes No
Monopod support test <5 seconds?	Yes No
Does the person live alone at home?	Yes No
Are there home caregivers?	Yes No
Does the person complain of memory problems?	Yes No
Does the person have prescriptions for more than 5 therapeutic classes on his/her prescription history for less than 6 months?	Yes No

For scores of 3 or more, the elderly patient was considered by our scale to be “frail.” For scores of 1 or 2, the patient was considered “pre-frail.” For a score of 0, the patient was considered “non-frail” or “robust.”

***Study population*** ***:*** *Our study population was made up of patients aged 75 or older who were monitored by a general practitioner and had an ADL (Activities of Daily Living) score of 4 or higher. Patients who did not provide their verbal consent during the introductory phase of the study, were under 75 years of age, had, or lived in nursing homes were excluded from the study.*

Patients less than 75 years old, an ADL score of less than 4, or whose primary caregiver could not be reached were excluded from this study. Those living in nursing homes were also excluded. Finally, patients who refused to participate in follow-up phone calls were not included. Information and Consent: Patient information was placed in the waiting rooms of general practitioners and/or consultation offices. It specified the name and type of work, the objective of the work, and its context. It also specified that patients could refuse to participate in the study without affecting the quality of their care.

*Study parameters* *:*

*Characteristics of the population*

The following data was collected:

- Gender
- Age
- Contact person: relationship and contact information
- ADL score
- Medical and surgical history
- Charlson comorbidity score: Charlson Comorbidity Index predicts the ten-year mortality for a patient who may have a range of comorbid conditions. The Charlson comorbidity index (CCI) is a commonly used scale for assessing morbidity (7).
- Height, weight, and BMI (Body Mass Index)

***Measuring frailty with Fried's criteria*** ***:*** *Fried’s scale (* 8 *) defines frailty on the basis of 5 criteria: fatigue, involuntary weight loss, reduced physical activity, slower walking speed, and decreased muscle strength. A point is assigned for each criterion, with patients considered “robust” or “non-frail” when none of the criteria are met, “pre-frail” when 1 or 2 of the criteria are met, and “frail” when 3 or more of the criteria are met.*

**Feasibility** **:** The feasibility of the “ZFS” tool for general medicine was determined by the amount of time that was required to screen for frailty. For the sake of fairness, one of the two methods was chosen at random to evaluate the patients. Patient Acceptability: Acceptability is a set of conditions that make this test acceptable to patients. It was measured using a visual analog, non graduated, scale that was appropriate for the question being asked. After using our ("ZFS") tool, patients were asked to rate their acceptability with this scale, between "completely unacceptable" and "completely acceptable." The instruction given was: "Place the cursor between these 2 options based on your opinion." 

The response was then weighted according to the same principle as the Visual Analog Scale (VAS) for pain: 

- Zero corresponded to "completely unacceptable" 

- Ten corresponded to "completely acceptable"

**Statistics** **:** The data was analyzed by XL-Stat software and collected anonymously using an EXCEL spreadsheet. First, a descriptive analysis of the results obtained was prepared. Quantitative variables were expressed as mean ± standard deviation and qualitative variables as absolute and relative numbers (percentages). Next, the "ZFS" tool was compared to Fried's criteria (which represents the gold standard) using the Student test for quantitative variables, and Chi² for qualitative variables. The ROC curve was calculated using GraphPad-Prism software. It is used to measure the performance of a diagnostic test and to determine optimal threshold values. 

 The relative risk of each item in the "ZFS" tool was estimated by calculating the Odds ratio, with a 95% confidence interval defined by the Miettinen method resulting in a 5% alpha probability. The “ZFS” score was assessed in terms of sensitivity, specificity, Youden's index, positive and negative predictive values, and the area under the ROC curve, using the Fried score as the gold standard. A Kappa coefficient was calculated to measure the concordance between the 2 tests.

As the Fried score provided 3 results (“frail,” “pre-frail,” and “non-frail”), we decided to consolidate the “pre-frail” and “robust” patients into one group (“non-frail”) for our study. We therefore compared the two scales, using the same groups for the “Zulfiqar Frailty Scale” tool. We reduced the number of types of patients from 3 to 2 to obtain a binary diagnosis of frailty (yes or no). A Pearson correlation matrix was used to evaluate discrepancies between the total scores and the items of each score. A paired two-sample t-test was used to compare the time it took to administer the 2 questionnaires. All the analyses were performed with R 4.0.2 software with an alpha risk set at 5%. 

Administrative Requirements: All patients who participated in our study were required to sign a consent form. We also obtained authorization to conduct the study from the Commission Nationale Informatique et Liberté (CNIL, “National Commission on Informatics and Liberty”, Poitiers, France). This data collection is declared to the CNIL, the French Data Protection Authority, at the Poitiers University of Medicine (86000 Poitiers, France, THESE.MEDECINE@univ-poitiers.fr ).

## Results

During our study, 200 patients were included. Overview: 103 women (51.5%) and 97 men (48.5%). 40% under 80 years of age. Mean age of 81.4±4.82 years (Table 2). Correlation between the two screening tools: Zulfiqar Frailty Scale and Fried’s criteria: In our study, there was a moderate correlation between the “ZFS” and the FRIED scores (r=0.65 [0.57; 0.73] 95% CI). Apart from a strong correlation between the 2 weight loss items (r=0.98), the correlation between the indicators of the 2 scores was weak, with a maximum r of 0.36 between the “slower walking speed” (FRIED) and “risk of falling” (ZFS) items (Table 3).

**Table 2:** Characteristics of the study population

General characteristics	
	Total patients ( = 200)
Age (years)	
Mean (standard deviation)	81.4 (4.82)
Median [min; max]	81.0 [75.0; 97.0]
75-79 years	80 (40)
80-84 years	64 (32)
85-89 years	41 (20.5)
90-94 years	14 (7)
95 years and over	1 (0.5)
Gender	
Female	103 (51.5 %)
Male	97 (48.5 %)
Weight (kg)	73.2 (18.7)
BMI* (kg/m²)	27.22 (5.14)
Types of medication	6.32 (3.6)
Charlson score (comorbidities score)	4.63 (1.01)
ADL*	5.81 (0.53)
IADL*	(1.58)

*BMI: Body Mass Index; *ADL: Activity Daily Living; *IADL: Instrumental Activity Daily Living.

**Table 3:** Pearson correlation between the items of the two tools: “ZFS” and Fried’s criteria

	Fried’s criteria				
Zulfiqar Frailty Scale	Weight loss	Fatigue/Exhaustion	Slower walking speed	Decreased muscle strength	Sedentariness
Weight loss	0.98	0.07	- 0.05	0.08	0.01
Balance on one leg	0.05	0.25	0.36	0.27	0.34
Lives alone	- 0.01	0.09	0.10	0.12	0.14
Home care	0.14	0.25	0.23	0.20	0.28
Memory loss	0.03	0.22	0.15	0.15	0.09
At least 5 different medications	0.06	0.27	0.35	0.20	0.34

*Descriptive analysis of the “ZFS” frailty screening tool* *: Using our new scale, we obtained a mean frailty score of 2.09±1.14. 70 patients (35.0%) were found to be “frail,” i.e. with a score of 3 or more on our screening scale (* Table 4 *).*

**Table 4:** Conditions measured with the “ZFS” tool

Zulfiqar Frailty Scale (ZFS)	Number of patients (N = 200)	%
Weight loss	29	14.5
Risk of falling	147	73.5
Lives at home alone	66	33.0
Presence of home care	61	30.5
Memory loss	115	57.5
Polymedicine	121	60.5

*Fried’s criteria* *: Using Fried’s criteria, 65 patients (32.5%) were found to be “frail,” i.e. with a score of 3 or higher. Of the remaining patients, 93 were considered “pre-frail” (46.5%), i.e. with a score of 1 or 2, and 42 were considered “non-frail” or "robust” (21.0%). For the purposes of our study, all these patients were assigned to the same group: “non-frail.” Using Fried's criteria, the mean frailty score was 1.90*±*1.36. Among our study population, there were significantly more women in the “frail” group (63.0%) than in the “non-frail” group (46.0%). In addition, each Fried criterion was a significant predictor of frailty (*Table 5*).*

**Table 5:** Comparison of Fried score items for frail and non-frail patients

	Frail (N = 65)	Non-frail (N = 135)	
Weight loss	19(29.0%)	9 (6.0%)	p<0.0001
Fatigue - Exhaustion	50(77.0%)	11 (8.0%)	p<0.0001
Slower walking speed	60(92.0%)	56(41.5%)	p<0.0001
Decreased muscle strength	51(78.0%)	47(35.0%)	p<0.0001
Decreased physical activity	45(69.0%)	29(21.5%)	p<0.0001

*Comparative analysis of the “ZFS” screening tool and Fried’s criteria* *.* Table 6 *. displays the full results of our screening tool using Fried’s criteria. 44 patients (67.7%) in the “frail” group according to Fried's criteria had a “Zulfiqar Frailty Scale” score of 3 or higher (out of 6), compared to 26 patients (19.3%) in the “non-frail” group (* Table 7 *). The difference between these two groups was significant (p<0.001).*

**Table 6:** Full summary of the results of the Zulfiqar frailty scale tool using Fried's criteria

Zulfiqar Frailty Scale (ZFS)	Frail (N = 65)	Non-frail (1) (N = 135)	p-value	Se (%)	NPV (%)	RR (95% CI)
Weight loss	19 (29.2%)	10 (7.4%)	< 0.001	29.2	73.0	2.44 (1.70; 3.50)
Balance on one leg	59 (90.3%)	88 (65.2%)	< 0.001	90.3	89.0	3.55 (1.63; 7.73)
Lives at home alone	27 (41.5%)	39 (28.9%)	0.08	41.5	72.0	1.44 (0.97; 2.14)
Presence of home care	35 (53.8%)	26 (19.3%)	< 0.001	53.8	78.0	2.66 (1.81; 3.90)
Memory loss	48 (73.8%)	67 (49.6%)	0.00131	73.8	80.0	2.09 (1.30; 3.36)
Polymedicine	54 (83.1%)	67 (49.6%)	< 0.001	83.1	86.0	3.21 (1.79; 5.74)

**Table 7:** Contingency table – Zulfiqar Frailty Scale vs. Fried’s criteria, with “pre-frail” and “robust” patients making up the “non-frail” group

	Fried’s criteria		
Zulfiqar Frailty Scale (ZFS)	Non-frail	Frail	Total
Non-frail	80 (40.0%)	8 (4.0%)	88 (44.0%)
Frail	55 (27.5%)	57 (28.5%)	112 (56.0%)
Total	135 (67.5%)	65 (32.5%)	200 (100%)

The “ZFS” tool had an AUC of 82.1 [76.13; 88.06] (95% CI) when compared with FRIED, after consolidating the "pre-frail” and “robust” patients into the “non-frail” group (Figure 2). A “ZFS” score of 3 or higher was most effective as a criterion for screening for frailty (sensitivity: 88.0% [77.0; 95.0] 95% CI, specificity: 59.0% [50.0; 68.0] 95% CI, Youden’s index: 47.0 [28.0; 62.0] 95% CI, positive predictive value: 51.0% [41.0; 60.0] 95% CI, negative predictive value: 91.0% [83.0; 96.0] 95% CI) (Table 8). Feasibility of the “ZFS” tool in general medicine: On average, it took 71.7 seconds (1 minute, 11 seconds, and 70 hundredths of a second) and 79.8 seconds (1 minute, 19 seconds, and 80 hundredths of a second) to administer the Zulfiqar Frailty Scale and Fried questionnaires, respectively. The Zulfiqar Frailty Scale questionnaire was therefore significantly faster (i.e., 8.14 (6.88; 9.40) seconds less (p < 0.001)). Our tool was very well-received by patients, with an acceptability rated at 9.8/10 using the visual analog scale.

**Figure 1:**
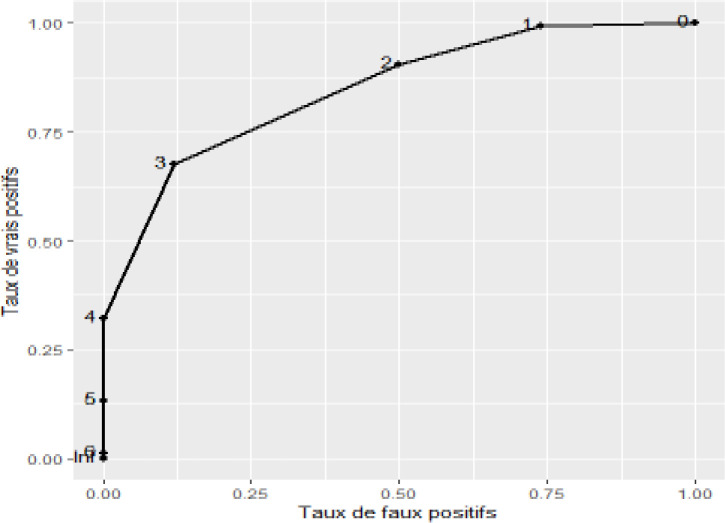
ROC curve, “ZFS” vs. FRIED score with consolidation of “pre-frail” and “robust” patients into one “non-frail” group

**Table 8:** Threshold values of the ROC curve

Threshold	Sensitivity (%)	Specificity (%)	Youden’s index (%)	Positive predictive value (%)	Negative predictive value (%)
> 0	100	9.0	9.0	35.0	100
> 1	97.0	25.0	22.0	38.0	94.0
> 2	88.0	59.0	47.0	51.0	91.0
> 3	58.0	90.0	49.0	75.0	82.0
> 4	25.0	96.0	21.0	76.0	73.0
> 5	3.0	100	3.0	100	68.0

## Discussion

We created a scale that can be used in primary care, one that takes clinical, psychological, and social factors into account so patients can be assessed in their entirety. The scale was made to be quick, simple, and efficient, with high sensitivity and negative predictive values. 

We wanted to evaluate the 6 items in our scale as effectively as possible, so decided to compare them to Fried's criteria. In our study, the prevalence of frailty, measured according to Fried's criteria, was 32.5%, or higher than expected. 

The “Zulfiqar Frailty Scale” tool has a sensitivity of 88.0%, making it comparable to other screening tool, and an outstanding negative predictive value of 91.0%, significantly reducing the risk of misdiagnosis or referring a patient to a team of specialists when such a referral is unnecessary.

Our tool has the advantage of not requiring any equipment whatsoever, making it perfectly suitable for primary care. The screening is quick and easy, allowing physicians to do away with time-consuming screening methods that inconvenience elderly patients. The time estimated for consultation in general medicine in France is about 15 minutes (9). With our scale, frailty screening could be assessed in 2 minutes, similar to the GFST scale. 

The time for performance is longer for the Fried Scale and for the SEGA grid A (from 6 to 10 minutes) (10), making them difficult to apply during consultation for general doctors. A practical tool should require minimal time to perform, similar to ours. In contrast to the Fried Scale, the ZFS does not require additional equipment such as a dynamometer for the determination of the isometric contraction. This is a real advantage in the context of large-scale screening. 

The goal of the "ZFS" tool was to harmonize professional practices and to make identifying frail members of the elderly population accessible during general practitioner visits. In practice, it should be offered to general practitioners to first implement preventive measures to limit complications related to frailty. Secondly, it can be used to refer patients to a geriatrician for a standardized geriatric assessment to establish a personalized care plan.

It is a quick tool with an average turnaround time of 71.7 seconds, that requires no prior training or specific equipment or facilities, making it a feasible screening tool suitable for general practice. Limitations: Our study sample remained small. To be validated for the purpose of studying its reproducibility, our tool must be tested in multiple general medicine practices, in urban and rural areas, and over a larger sample with many types of practitioners (doctors, nurses, physical therapists, occupational therapists). The prediction of pathological events (falls, hospitalization, and morbidity-mortality) was not studied in this research. This task will start in March 2021, for an 18-month period. The cognitive question remains difficult to understand with a rapid detection score such as ours. The item, “Does the person complain of memory problems?” is still subjective and requires, as we have done, a response confirmed by the patient's family.

**In conclusion** **our scale must be tested further in other general practices by recruiting a wider range of participants. Eventually, the reproducibility and ability of the scale to predict potentially dangerous situations must be developed and tested on elderly patients, which will take place in the upcoming weeks and months.**
